# Filling the gap between biology and computer science

**DOI:** 10.1186/1756-0381-1-1

**Published:** 2008-07-17

**Authors:** Jesús S Aguilar-Ruiz, Jason H Moore, Marylyn D Ritchie

**Affiliations:** 1 School of Engineering, Pablo de Olavide University, Seville, Spain; 2 Norris-Cotton Cancer Center, Lebanon, New Hampshire, USA; 3 Dartmouth Medical School, Lebanon, New Hampshire, USA; 4 Center for Human Genetics Research, Vanderbilt University, Nashville, TN, USA

## Abstract

This editorial introduces *BioData Mining*, a new journal which publishes research articles related to advances in computational methods and techniques for the extraction of useful knowledge from heterogeneous biological data. We outline the aims and scope of the journal, introduce the publishing model and describe the open peer review policy, which fosters interaction within the research community.

## Aim and scope

*BioData Mining *[1] is an open-access, open peer-reviewed, online journal that publishes articles on the development of data mining techniques applied to biological data. The journal stems from the gap between biology and computer science and covers a number of topics in the middle of these fields. One of the main interests of *BioData Mining *is the advance in computational methods or theoretical informatics for the progress in the discovery of new knowledge in biomedical sciences.

Data mining [2] techniques have been traditionally used in many varied contexts. Usually datasets contained many examples (thousands) and some attributes (at most several tens). Algorithms have been developed taking into account these characteristics, and have been validated by means of statistical tests with synthetic and real-world data. Statistics has been the support for any analysis of biological data for many years. However, the biological data has changed over time in size, but above all in structure, and many challenges arise from genetic, transcriptomic, genomic, proteomic and metabolomic data.

The enormous increase of biological data incorporates another element of difficulty because statistics, without losing its relevance, has moved to the background leaving in the foreground a space for complex heuristics. In addition, the curse of dimensionality plays an important role in the design of new data mining algorithms. However, the most important challenge comes from the intrinsic characteristics of new problems to be solved. Due to the high volume of data, optimization and efficiency are key aspects in the design of new heuristics, which many times only provide approximate solutions.

In this sense, *BioData Mining *aims at publishing articles that not only adapt, evaluate or apply traditional data mining techniques, but also that develop, evaluate or apply novel methods from data mining or machine learning fields to the analysis of complex biological data.

Moreover, the situation has substantially changed during the last decade. Nowadays, biological information is distributed and adopts different formats. It is not trivial to consider different types of data, which are located in different databases and present various levels of structure or heterogeneity. In some cases the effort is focused on facilitating the management of biological information, dealing with semantic aspects of the information through the Internet.

In order to promote the advance in science many research groups are making their software development projects publicly available, as open-source software, which encourages researchers to develop extensions of verified software applications, like interfaces, packages or specific services.

*BioData Mining *aims at publishing articles that design, develop and integrate databases, software and web services for the storage, management and retrieval of complex biological data, with emphasis on open-source software for the application of data mining to the analysis such type of information.

The role of biologists, geneticists, physicians, etc. is critical in the correct interpretation of results obtained by data mining algorithms. In many cases, data needs to be pre-processed for extracting useful knowledge and, in some cases, algorithms produce models that must be post-processed to get an insight of the knowledge that information hides. At the end, experimental validation is crucial to show the research community the quality of the approaches. In this field, statistics offers robust tools that can be applied directly, although new developments are also needed to deal with biological data.

*BioData Mining *aims at publishing articles that present new methods for pre-processing, post-processing and validation of data mining algorithms for the analysis of genetic, transcriptomic, genomic, proteomic, and metabolomic data.

In the expectation of filling the gap between biology and computer science, we believe that BioData Mining will contribute to the development of theoretical and practical aspects of new methodologies driven by biological data.

## Open access and open peer review publishing model

The time interval between the date an article is written and the date an article is read should be as short as possible. Long intervals are mainly due to slow reviewing process and limited access to articles. *BioData Mining *will put much effort into reducing the reviewing process to several weeks, and will avoid the other aspect due to the open access nature of the journal, i.e., articles will be fully accessible online to any reader immediately upon publication.

In order to make the peer review process transparent *BioData Mining *has adopted an open-review policy. Reviewers' names are included on the peer review reports and are made publically available upon acceptance of an article. We believe that this will foster constructive reviews, and therefore enrich the criticism. This policy will contribute greatly in driving young researchers to improve the quality of their articles.

During the last years, many journals have adopted the open-access policy. Nowadays the success is unquestionable. We expect that the open peer review policy will follow a similar path in the near future, and some experiences show enthusiasm for the concept, such as PLoS ONE[3], that strongly urge reviewers to relinquish the anonymity to promote open decision-making.

Finally, to facilitate the search for topics or related research in articles published in *BioData Mining*, the readers will find all the articles archived in PubMed Central [4].

## Editorial Board

The journal is run by two Editors-in-Chief, who subscribe this editorial. Marylyn D. Ritchie acts as Managing Editor. The members of the Editorial Board cover a wide range of research fields related to Biology and Computer Science. To mention some expertise, it ranges from Biomedical Informatics (M. Ramoni [5], F. Azuaje [6]) to Structural Bioinformatics (R. Casadio [7], D. Jones [8], J. M. Carazo [9]), from Soft Computing (O. Cordon [10], I. Zwir [11]) to Clinical Research (M. Eppstein [12]), from Machine Learning (E. Marchiori [13], K. Cios [14]) to Evolutionary Computation (P. Larrañaga [15]), from Cancer Research (S. Volinia [16]) to Data Mining (J. Aguilar-Ruiz [17], D. Simovici [18], D. Gamberger [19], H. Toivonen [20]), from Biostatistics (J. Rahnenfuhrer [21]) to High–performance Technologies (R. Schneider [22]), from Immunology (B.A. McKinney [23]) to Computational Genetics (J.H Moore [24], M.D. Ritchie [25]), from Database Integration (M. Kanehisa [26]) to Functional Genomics (S. Kasif [27]), from Software Technologies (A. Omicini [28]) to Stem Cells (B. Soria [29]).
